# Three days of a eucaloric, low-carbohydrate/high-fat diet increases insulin clearance in healthy non-obese Japanese men

**DOI:** 10.1038/s41598-019-40498-6

**Published:** 2019-03-07

**Authors:** Ruriko Suzuki, Yoshifumi Tamura, Kageumi Takeno, Saori Kakehi, Takashi Funayama, Yasuhiko Furukawa, Hideyoshi Kaga, Daisuke Sugimoto, Satoshi Kadowaki, Yuki Someya, Akio Kanazawa, Ryuzo Kawamori, Hirotaka Watada

**Affiliations:** 1 Department of Metabolism & Endocrinology, Juntendo University Graduate School of Medicine, Tokyo, Japan; 2 Sportology Center, Juntendo University Graduate School of Medicine, Tokyo, Japan; 3 Center for Therapeutic Innovations in Diabetes, Juntendo University Graduate School of Medicine, Tokyo, Japan; 4 Center for Identification of Diabetic Therapeutic Targets, Juntendo University Graduate School of Medicine, Tokyo, Japan

## Abstract

Metabolic clearance rate of insulin (MCRI) is thought to help maintain glucose homeostasis even in healthy subjects. However, the effect of a low carbohydrate/high fat (LCHF) diet on MCRI in healthy subject remains unclear. To investigate the effect of a 3-day eucaloric LCHF diet on MCRI in healthy subjects, we studied 42 healthy non-obese Japanese men. Each subject consumed a eucaloric LCHF diet for 3 days. Before and after the LCHF diet, intramyocellular lipid (IMCL) levels were measured using ^1^H-magnetic resonance spectroscopy, and glucose infusion rate (GIR) and MCRI were evaluated with a euglycemic hyperinsulinemic clamp. The LCHF diet increased MCRI by 10% and decreased steady state serum insulin (SS_SI_) and GIR during glucose clamp by 10% and 6%, respectively. To further investigate the role of MCRI, we divided subjects into high-responder (HR) and low-responder (LR) groups based on the median %change in MCRI. The LCHF diet increased IMCL and decreased SS_SI_ during glucose clamp in the HR group, while those were not altered in the LR group. Our results suggested that a 3-day eucaloric LCHF diet increases MCRI in healthy non-obese Japanese men. This change seemed to be beneficial in terms of maintaining euglycemia during low carbohydrate availability.

## Introduction

Insulin resistance and hyperinsulinemia are often observed in unhealthy obese subjects^1^. Hyperinsulinemia to counter insulin resistance is caused by both enhanced insulin secretion and decreased insulin clearance. Decreased insulin clearance could be considered as a regulatory mechanism for maintaining euglycemia when insulin sensitivity is impaired^2^. We recently found that impaired metabolic clearance rate of insulin (MCRI) was observed even in non-obese healthy subjects with modest insulin resistance and could be considered to be a compensatory phenomenon for maintaining glucose uptake and metabolic status in the face of slightly impaired insulin sensitivity in muscle^3^. However, how MCRI is regulated in healthy subjects is not fully understood.

Insulin sensitivity is impaired during weight gain by a high-calorie, high-fat (HF) diet, for which hyperinsulinemia can be compensatory. One week of a high-calorie, HF diet in healthy subjects impairs insulin sensitivity and increases insulin levels. This increase in insulin levels is mainly induced by decreased MCRI rather than enhanced insulin secretion^4^. Thus, a high-calorie, HF diet is considered to decrease MCRI^2^. However, the effect of an HF diet on MCRI might vary by ethnicity and dietary protocol. Indeed, 5 days of a high-calorie, HF diet increases MCRI in South Asians, but the same diet protocol does not alter MCRI in Caucasians^5^. In other studies, a eucaloric, low-carbohydrate, high-fat (LCHF) diet did not alter MCRI in Caucasians and African-American^6–8^. These data suggest that the effect of an HF diet on MCRI has not been elucidated yet, and that the effect of an LCHF diet on MCRI in East Asians has not been investigated yet.

As a result, the present study was designed to investigate the effect of a 3-day eucaloric LCHF diet in non-obese healthy men. For this purpose, we recruited healthy non-obese Japanese men in whom we measured insulin clearance and glucose infusion rate (GIR) using a euglycemic hyperinsulinemic clamp.

## Materials and Methods

### Study subjects

The study subjects consisted of 42 healthy non-obese male volunteers (Table 1). They were in good health as determined by a medical history, physical examination, and fasting glucose and lipid levels (Table 1). All subjects gave written informed consent for the study, which was approved by the Ethics Committee of Juntendo University. This study was carried out in accordance with the principles outlined in the Declaration of Helsinki.

**Table 1:** Various clinical parameters before and after a 3-day eucaloric low-carbohydrate high-fat (LCHF) diet.

	Pre	Post	P value
n	42	—	—
Age (years)	23 (22–25)	—	—
BMI (kg/m^2^)	22.7 (20.8–24.3)	22.4 (20.8–24.2)	<0.01
Total body fat content (%)	16.0 (13.7–19.6)	16.3 (13.5–18.6)	0.070
Daily Physical Activity (kcal/day)	275.3 (223.5–327.8)	274.7 (225.7–332.1)	0.224
Fasting plasma glucose (mg/dl)	86.5 (84.0–90.0)	86.5 (83.0–90.0)	0.391
Fasting serum insulin (μU/mL)	3.89 (3.12–4.46)	3.14 (2.45–4.23)	<0.01
Fasting serum C-peptide (ng/ml)	1.12 (0.90–1.35)	0.95 (0.74–1.15)	<0.01
Free fatty acid (μEq/L)	405 (350–520)	423 (357–473)	0.750
Triglyceride (mg/dl)	71 (51–87)	38 (28–49)	<0.01
HDL-C (mg/dl)	52 (46–59)	52 (47–61)	0.527
HbA1c (%)	4.7 (4.6–4.8)	4.7 (4.6–4.8)	1.000
High molecular weight-adiponectin (ng/ml)	1.37 (0.75–1.60)	1.21 (0.83–1.51)	0.019
Acetoacetic acid	31 (22–45)	73 (53–107)	<0.01
3-Hydroxybutyric acid	64 (35–115)	161 (109–208)	<0.01
Total ketone body	92 (59–154)	242 (159–311)	<0.01
VO_2peak_ (ml/kg per min)	47.8(43.5–55.1)	—	—
Intramyocellular lipid in TA (S-fat/Cre)	2.4 (1.4–3.1)	2.6 (2.0–3.9)	<0.01
Intramyocellular lipid in SOL (S-fat/Cre)	7.6 (5.8–10.2)	9.2 (6.6–12.4)	<0.01
Metabolic clearance rate of insulin (ml/min per m^2^)	494.4 (463.2–548.0)	542.4 (497.7–571.2)	<0.01
Steady state serum insulin (μU/mL)	206.5 (185.3–218.8)	186.8 (176.1–202.8)	<0.01
Glucose infusion rate (mg/kg FFM·min^−1^)	12.0 (10.8–13.6)	11.3 (10.0–12.9)	0.02
Glucose infusion rate (mg/kg FFM·min^−1^)/Log_10_ Steady state serum insulin (μU/mL)	5.20 (4.61–5.99)	4.96 (4.42–5.66)	0.07

Data are median (IQR). TA; tibialis anterior muscle, SOL; soleus muscle, S-fat; methylene signal intensity, VO_2peak_; peak oxygen consumption.

### Study design

This study is a sub-analysis of our previous research study investigating the effect of a 3-day, eucaloric LCHF diet on insulin sensitivity and ectopic fat in muscle^9,10^. In those previous studies, we did not assess MCRI, thus, we evaluated available insulin clearance data in the current study. Briefly, the study subjects were prohibited from regular exercise from 7 days before dietary intervention to the end of the study. Seven days before dietary intervention, the mean daily physical activity level was estimated using an ambulatory accelerometer. Biochemical analyses of serum samples and total body fat content were analyzed as described previously^9^.

### Dietary manipulation

During the intervention period, subjects were provided with packed meals prepared by a food company (Musashino Foods, Saitama, Japan). Each subject was provided a weight-maintaining, eucaloric, normal-fat diet (25% fat, 55% carbohydrate, 20% protein) for 3 days, followed by an LCHF diet (60% fat, 20% carbohydrate, 20% protein) for 3 days^9^. We asked subjects to consume only the menu we provided, and the dietary compliance was monitored by self-diary. Previous study suggested that increased consumption of saturated fatty acids, but not unsaturated fatty acids, is associated independently with insulin resistance^11^. In addition, it has been shown that the amount of saturated fatty acids in intramyocellular TG is key determinants of insulin resistance^12^. For these reasons, we set the fat composition of the LCHF diet as $\sim$45% saturated, $\sim$30% monounsaturated, and $\sim$25% polyunsaturated fatty acid.

### Euglycemic hyperinsulinemic glucose clamp

The euglycemic hyperinsulinemic glucose clamp study (target plasma glucose level of 95 mg/dL and insulin infusion rate of 100 mU/m^2^·min) was performed using an artificial pancreas (STG22; Nikkiso, Shizuoka, Japan), as reported previously^13^. The steady-state GIR was observed from 105 to 120 min after the beginning of the study^14,15^. When circulating insulin level is very high (e.g. more than ~160 μU/ml), the relationship between log transformed insulin (Log-insulin) and GIR is seemed to be linear^16^. Thus, we also preliminary calculated corrected GIR by log transformed steady-state serum insulin (SS_SI_) during glucose clamp (GIR/Log-SS_SI_).

### Calculation of metabolic clearance rate for serum insulin (MCRI)

MCRI during glucose clamp was calculated using the following equation: MCRI = {IIR/[SS_SI_ − (B_SI_ * SS_SC_/B_SC_)]}, where IIR = insulin infusion rate, SS_SI_ = steady-state serum insulin during glucose clamp, B_SI_ = basal serum insulin, SS_SC_ = steady-state serum C-peptide during glucose clamp, B_SC_ = basal serum C-peptide^17^. C-peptide data were included to account for the suppression of endogenous insulin secretion by exogenous insulin.

### ^1^H-magnetic resonance spectroscopy (MRS)

Intramyocellular lipid (IMCL) values for the right tibialis anterior (TA) and soleus (SOL) were measured using ^1^H-MRS (VISART EX V4.40, Toshiba, Tokyo, Japan)^14,15^. After ^1^H-MRS measurements, IMCL was quantified using methylene signal intensity (S-fat) with the creatine signal (Cre) as the reference^14,15^.

### Statistical analysis

Data are presented as median (interquartile range: IQR). The Wilcoxon signed rank test was used for comparison of paired observations. Differences between two groups were compared using the Mann-Whitney U-test. The group × treatment interaction was analyzed using 2-way repeated ANOVA followed by Bonferroni’s post hoc test. Statistical significance was set at P < 0.05.

## Results

### Characteristics of subjects and changes in metabolic parameters after a eucaloric, low-carbohydrate, high-fat (LCHF) diet

All subject, except 2 subjects, completely consumed all the specified LCHF meals. Appetite in 2 subjects slightly decreased during the LCHF diet, however, they consumed 96% or 97% of the specified meals for each. Table 1 shows the effects of dietary intervention on various parameters. Fasting plasma glucose and free fatty acid levels did not change after dietary intervention. Insulin and triglyceride levels significantly decreased after the eucaloric LCHF diet. Ketone body levels increased, while BMI and high-molecular-weight adiponectin levels decreased slightly after the eucaloric LCHF diet. In addition, TA and SOL IMCL levels increased significantly and GIR decreased significantly after the eucaloric LCHF diet. However, when GIR is adjusted by Log-SS_SI_, the P value for the difference becomes not significant (P = 0.07) (Table 1).

### Characteristics of subjects in the high-responder (HR) and low-responder (LR) groups

As shown in Table 1, the eucaloric LCHF diet decreased MCRI, however it was highly variable across individuals. Thus, to further investigate the factors associated with interindividual variations in MCRI after the LCHF diet, we classified subjects as high-responder (HR) (subjects whose %change in MCRI after the LCHF diet was greater than the median) and low-responder (LR) (subject whose %change in MCRI after the LCHF diet was less than the median).

As shown in Table 2, baseline parameters were similar between the two groups. In 2-way repeated ANOVA analysis, the group × treatment interactions in IMCL in TA and SS_SI_ were significant. Post hoc analysis revealed that IMCL in TA were increased and SS_SI_ was decreased in the HR group, while those were not changed in the LR group.

**Table 2:** Clinical characteristics of the High-responder (HR) group and the Low-responder (LR) group.

	Low-responder (LR)		High-responder (HR)		P value for two-way repeated ANOVA
	Pre	Post	Pre	Post	
n	21	—	21	—	—
Metabolic clearance rate of insulin (ml/min per m^2^)	508.5 (460.9–569.7)	498.1 (453.0–527.6)	492.7 (464.1–499.6)	561.8 (547.1–627.8)	—
Age (years)	23 (21–24)	—	23 (22–25)	—	—
BMI (kg/m^2^)	22.5 (19.8–24.4)	22.2 (19.4–24.2)	22.8 (21.2–24.1)	22.4 (21.1–24.0)	0.11
Fasting plasma glucose (mg/dl)	87 (86–90)	87 (83–90)	86 (83–91)	85 (83–90)	0.68
Fasting serum insulin (μU/mL)	3.85 (3.05–4.28)	3.15 (2.44–4.57)	4.13 (3.30–4.67)	3.13 (2.50–3.75)	0.83
Fasting serum C-peptide (ng/ml)	1.13 (0.91–1.31)	0.99 (0.81–1.21)	1.11 (0.90–1.35)	0.92 (0.72–1.12)	0.72
Free fatty acid (μEq/L)	389 (348–539)	419 (360–480)	410 (360–470)	430 (340–460)	0.97
Triglyceride (mg/dl)	71 (49–79)	35 (29–46)	70 (52–100)	40 (28–49)	0.52
HbA1c (%)	4.7 (4.6–4.8)	4.7 (4.6–4.7)	4.6 (4.5–4.8)	4.7 (4.5–4.8)	0.06
High molecular weight-adiponectin (ng/ml)	1.45 (0.96–1.87)	1.29 (0.96–1.60)	1.05 (0.70–1.45)	1.16 (0.82–1.34)	0.55
Intramyocellular lipid in TA(S-fat/Cre)	2.4 (1.4–3.3)	2.1 (2.0–3.1)	2.3 (1.3–3.1)	3.0 (2.5–4.4) *	<**0**.**05**
Intramyocellular lipid in SOL (S-fat/Cre)	8.0 (7.1–11.5)	9.2 (6.5–11.3)	6.6 (4.9–9.3)	9.6 (7.1–12.7)	0.08
Total body fat content (%)	15.7 (13.8–20.7)	16.3 (12.1–19.9)	17.5 (13.6–18.7)	16.2 (13.6–18.1)	0.18
VO_2peak_ (ml/kg per min)	47.0 (43.0–52.7)	—	47.8 (43.5–55.9)	—	—
Daily Physical Activity (kcal/day)	256 (222–324)	272 (212–326)	278 (257–329)	279 (239–334)	0.59
Acetoacetic acid	26 (19–43)	68 (50–110)	34 (24–54)	76 (63–68)	0.79
3-Hydroxybutyric acid	59 (33–107)	162 (73–206)	64 (40–115)	161 (120–208)	0.94
Total ketone body	84 (57–146)	228 (130–311)	98.5 (70–167)	242 (175–300)	0.88
Steady state serum insulin (μU/mL)	200 (177–219)	202 (192–223)	208 (201–218)	180 (161–186) *	<**0**.**05**
Glucose infusion rate (mg/kg FFM·min^−1^)	11.7 (10.6–13.0)	11.4 (10.3–12.4)	12.7 (11.0–14.0)	11.2 (10.0–13.2)	0.10
Glucose infusion rate (mg/kg FFM·min^−1^)/Log_10_ Steady state serum insulin (μU/mL)	5.15 (4.55–5.78)	4.93 (4.40–5.43)	5.51 (4.71–6.17)	5.00 (4.47–6.01)	0.45

Data are median (IQR). TA; tibialis anterior muscle, SOL; soleus muscle, S-fat; methylene signal intensity, VO_2peak_; peak oxygen consumption.

^*^P < 0.05 vs. Pre in HR group.

## Discussion

In the present study, we investigated the effect of a eucaloric LCHF diet on MCRI in healthy non-obese Japanese men. Although previous studies have suggested that a eucaloric LCHF diet does not alter MCRI in Caucasians and African-Americans^6–8^, it significantly increased MCRI in the present study. While individual changes in MCRI were highly variable, the subjects with greater change in MCRI with the eucaloric LCHF diet were characterized by decreased SS_SI_ and increased IMCL accumulation after the LCHF diet.

The mechanisms by which MCRI increases after the eucaloric LCHF diet are currently unknown. However, decreases in insulin concentration as a result of increased MCRI after the eucaloric LCHF diet is considered as a reasonable biological reaction in terms of glucose homeostasis, because a LC diet decreases carbohydrate availability^18^. In this regard, it has been reported that patients with anorexia nervosa have lower fasting insulin and glucose levels and increased MCRI; however, these changes were normalized after successful treatment^19^. In addition, sodium glucose cotransporter (SGLT) 2 inhibitors also increase MCRI^20^. Acutely, SGLT2 inhibitors enhance both urinary glucose excretion and insulin clearance, suggesting a potential link between glucose loss and increased insulin clearance. However, the effect of chronic SGLT2 inhibitor use might be due to weight loss. Further studies are required to elucidate the relationship between carbohydrate availability and MCRI.

In the present study, GIR decreased significantly after the eucaloric LCHF diet. However, SS_SI_ was also significantly decreased after the LCHF diet, thus this change might contribute to decreased GIR. Thus, we adjusted GIR by Log-SS_SI_ (GIR/Log-SS_SI_)^16^ and found that the P value for the difference becomes not significant (P = 0.07) (Table 1). This potentially suggested that decreased SS_SI_ after the LCHF diet might partly contribute to decreased GIR.

The effect of a HF diet on MCRI might vary by ethnicity and dietary protocol. Since insulin resistance is closely associated with decreased MCRI in obese subjects^1,21,22^, MCRI is expected to be impaired in parallel with the development of insulin resistance during weight gain. However, 5 days of a high-calorie, HF diet increased MCRI and decreased insulin sensitivity in South Asians^5^, whereas both parameters were unchanged in Caucasians. These metabolic changes seen in South Asians are similar to our data showing a eucaloric LCHF diet simultaneously increases MCRI and decreases GIR. In addition, a few studies have reported that a similar eucaloric LCHF diet did not change MCRI in Caucasians and African-Americans^6–8^. Thus, we speculate that increased MCRI and decreased insulin sensitivity occurring after a HF diet is specific to Asians, regardless of caloric intake.

In this study, we divided subjects into HR and LR groups based on the median %change in MCRI after the LCHF diet and compared these phenotypes. After the LCHF diet, decreased SS_SI_ and IMCL accumulation in TA occurred concomitantly in the HR group only; however, the underlying mechanisms linking MCRI and IMCL changes are totally unknown. In addition, there were no significant differences in baseline data between the HR and LR groups. Thus, we cannot even speculate on why the subjects had different responses to the eucaloric LCHF diet, except for differences in MCRI. Future studies using a genetic approach and a biological approach with muscle and liver samples would be needed to identify this variability.

Our study has several limitations. We recruited only men because men have higher risk for metabolic and cardiovascular diseases than women^23^. However, the sex differences in body fat distribution^24^ and IMCL utilization^25^ have been shown previously. Therefore, it is unclear if our data can be applied to women. In addition, we did not investigate baseline dietary intake of each subject before intervention. Although we provided normal fat diet for 3-day to decrease the effect of previous dietary intake, we cannot deny the possibility that previous dietary composition influenced on the results.

In conclusion, 3 days of a eucaloric LCHF diet increased MCRI in healthy non-obese Japanese men. In addition, individual changes in MCRI were highly variable across individuals. HR subjects were characterized by IMCL accumulation and decreased SS_SI_ after the eucaloric LCHF diet. Although the mechanism underlying this response is unclear, these physiological changes seemed to be beneficial in terms of maintaining euglycemia in insulin-sensitive subjects.
